# Identification and Characterization of 24-Dehydrocholesterol Reductase (DHCR24) in the Two-Spotted Cricket, *Gryllus bimaculatus*

**DOI:** 10.3390/insects12090782

**Published:** 2021-09-01

**Authors:** Yin Shan Isa Mack, Masatoshi Dehari, Nobukatsu Morooka, Shinji Nagata

**Affiliations:** 1 Department of Integrated Biosciences, Graduate School of Frontier Sciences, The University of Tokyo, Chiba 277-8562, Japan; 9247422196@edu.k.u-tokyo.ac.jp (Y.S.I.M.); masa10toshi16@gmail.com (M.D.); 2 Institute for Molecular and Cellular Regulation, Gunma University, Maebashi 371-8512, Japan; n.morooka@gmail.com

**Keywords:** DHCR24, desmosterol, cholesterol, phytosterols, cricket, omnivorous insects, RNAi

## Abstract

**Simple Summary:**

DHCR24 (24-dehydrocholesterol reductase) is a key enzyme for producing cholesterol from desmosterol and that is also involved in the conversion of plant sterols to cholesterol in most plant-feeding insects. This study extensively examined the possibility of DHCR24 involved in the sterol conversion in omnivorous insects, which feed on multiple food origins. Homologs of DHCR24 (GbDHCR24-1 and -2) were identified and characterized by using the two-spotted cricket, *Gryllus bimaculatus*, as an experimental model. The quantitative expression analyses and RNA interference experiments revealed that GbDHCR24-1 rather than GbDHCR24-2 facilitates the desmosterol-to-cholesterol conversion in crickets. Our data suggested that the omnivorous species produced cholesterol from desmosterol in the same manner as the plant-feeding species do.

**Abstract:**

Arthropods, including insects, convert sterols into cholesterol due to the inability to synthesise cholesterol de novo. 24-dehydrocholesterol reductase (DHCR24) plays an important role in the conversion. Not only involving the cholesterol biosynthesis in vertebrates, DHCR24 is required for the conversion of desmosterol into cholesterol in phytophagous insects. The current study extensively examined DHCR24 in omnivorous insects, which feed on both plants and animals, using *Gryllus bimaculatus* as the experimental model. We identified cDNAs encoding two homologues of DHCR24 from *G. bimaculatus*, which were designated as GbDHCR24-1 and GbDHCR24-2. Both homologues contained the flavin adenine dinucleotide binding domain, which is a feature of DHCR24. Quantitative polymerase chain reaction revealed that among tissues of adult crickets, fat body and anterior midgut expressed high levels of GbDHCR24s. Both fat body and anterior midgut demonstrated DHCR24 activities in which one of the functions is the conversion of desmosterol into cholesterol in vitro. Knockdown of GbDHCR24-1 significantly reduced the conversion activity in the anterior midgut while knockdown of the GbDHCR24-2 did not. Additionally, the accumulation of desmosterol was detected in a feeding experiment with a specific DHCR24 inhibitor, azacosterol. We finally concluded that GbDHCR24-1 is the major enzyme that facilitates the desmosterol-to-cholesterol-conversion in crickets.

## 1. Introduction

Cholesterol is critical for the survival of many organisms because of its varied functions. In the cholesterol biosynthesis of vertebrates, two pathways, which are known as the Bloch pathway and the Kandutsch-Russell pathway, are involved [1]. In both pathways, an enzyme called 24-dehydrocholesterol reductase (DHCR24) reduces Δ24 in sterols. In mouse and other mammals, DHCR24 converts desmosterol to cholesterol in the Bloch pathway while DHCR24 converts lanosterol to 24,25-dihydrolanosterol in the Kandutsch–Russell pathway [2,3,4]. Despite arthropods, including insects, being unable to synthesise cholesterol de novo, DHCR24 is equally important to them [5]. Insects, particularly phytophagous insects that feed on plants convert plant sterols (phytosterols), such as β-sitosterol, campesterol and stigmasterol, into cholesterol to meet their cholesterol requirement [6]. The phytosterols are converted into desmosterol via dehydration, epoxidation and dealkylation [6,7,8,9,10,11,12]. Desmosterol is then converted to cholesterol. In the silkworm, *Bombyx mori*, DHCR24 has been shown to have the potential to convert desmosterol into cholesterol in vitro [13,14]. There are two DHCR24 variants in *B. mori*. Both are expressed in endoplasmic reticulum (ER) of larval midgut [15]. One homologue, BmDHCR24-1, shows significant enzyme activity, but the other homologue, BmDHCR24-2, exhibits almost no activity. The conversion is also facilitated by DHCR24 in other species, such as *Caenorhabditis elegans* [16].

Although phytophagous insects have been proven to use DHCR24 to convert desmosterol to cholesterol, it is still unclear if insects feeding on meat and other types of food exhibit this enzyme activity. Omnivorous insects, which feed on both herbivorous and carnivorous diets, may be able to catalyse more types of sterols than species that only feed on one type of food. Since omnivorous insects also feed on plants, they are very likely to metabolise phytosterols such as the phytophagous insects do, but it is still uncertain if the phytosterol conversion pathways in the omnivorous and phytophagous insects are exactly the same [5]. In the present study, we used the two-spotted cricket, *Gryllus bimaculatus*, as an experiment model to investigate the conversion of desmosterol in omnivorous insects. We first identified the putative DHCR24 sequences in crickets. Then, we examined the enzyme activity of DHCR24. Through the knockdown of DHCR24 variants, we investigated the activity of each variant. Moreover, we used a specific DHCR24 inhibitor, azacosterol (also known as 20,25-diazacholesterol), to examine if DHCR24 would be the only enzyme that was capable of converting desmosterol to cholesterol in crickets [17].

## 2. Materials and Methods

### 2.1. BLAST Search and Phylogenetic Analysis

Two amino acid sequences of *Bombyx mori* DHCR24, BmDHCR24-1 and BmDHCR24-2 were used to search *Gryllus bimaculatus* cDNA sequences encoding DHCR24 homologues with our in-house database generated from RNA-sequencing analyses [13,14]. An alignment of amino acid sequences of *B. mori* DHCR24s and putative amino acid sequences in *G. bimaculatus* DHCR24s were generated using Clustal W. Phylogenetic analysis was performed with MEGA X. The phylogenetic tree was constructed by the Neighbour Joining method with 1000 bootstrap replicates. 7-dehydrocholesterol reductase in *Homo sapiens* was used as an outgroup in the phylogenetic analysis.

### 2.2. Experimental Insects

The two-spotted cricket, *G. bimaculatus*, was reared in plastic boxes with water supply and powder grounded from rabbit food (LRC4, Oriental Yeast Co., Ltd., Tokyo, Japan) and cat food (Carat Mix (fish flavour), Petline, Japan) (1:4 *w*/*w* ratio). Crickets were reared at 28 ${}^{\circ }$C with a relative humidity of approximately 40% in a 16 h light and 8 h dark photoperiod regime.

### 2.3. Preparation of Azacosterol Diet

The diet was prepared by mixing rabbit food (LRC4) and azacosterol (AdooQ BioScience, Irvine, CA, USA) dissolved in dichloromethane (Wako, Osaka, Japan). After evaporating dichloromethane to dryness, crickets were given the azacosterol-rabbit diet of a final concentration of 20 ppm (*w*/*w*) for 18 h and were kept individually.

### 2.4. Preparation of Ultrancentrifugal Fractions

Fat body and anterior midgut were dissected out from 8–10 adult females on the second day after adult emergence using 0.9% NaCl aqueous solution containing 0.1% (*v*/*v*) 200 mM 4-amidinophenylmethanesulfonyl fluoride hydrochloride (*p*-AMPSF) (Wako, Osaka, Japan) in dimethyl sulfoxide. The anterior midguts were submerged in the homogenising solution (20 mM 4-(2-hydroxyethyl)-1-piperazineethanesulfonic acid (HEPES), 0.66 mM MgCl${}_{2}$, pH 7.4) containing cOmplete Mini EDTA-free protease inhibitor cocktail (Sigma Chemical Company, Tokyo, Japan) and were homogenised by the glass Teflon homogenisers. Four ultracentrifugal fractions (7700× *g*-pellet; 15,300× *g*-pellet; 116,200× *g*-pellet; final supernatant) in the homogenates were isolated using the differential centrifugation method adapted by Liebrich and Hoffmann with slight adjustments [18]. The pellets were resuspended in the reaction solution (100 mM HEPES, 3.3 mM MgCl${}_{2}$, pH 7.5) containing 50 µM *p*-AMPSF. The protein concentration of each ultracentrifugal fraction was determined using the Bradford reagent (Bio-Rad, Hercules, CA, USA), and the absorbance was measured at 595 nm. Solutions of known concentrations of bovine serum albumin (BSA) (Sigma Chemical Company, Japan) were used as standards.

### 2.5. Evaluation of Enzyme Activity

A substrate solution (solution S) composed of 1 mg/mL of deuterated desmosterol (desmosterol-d6) (Avanti Polar Lipids, Alabaster, AL, USA) in 45% (2-hydroxyethyl)-β-cyclodextrin dissolved in water was prepared. An incubation mixture with a total volume of 100 µL composed of 1 µL of solution S, 3.5 mM of NADPH, and reaction solution containing 50 µM *p*-AMPSF was incubated with one ultracentrifugal fraction containing 10 µg of protein at 37 ${}^{\circ }$C for 16–18 h. The sterols were extracted from the incubation mixture with *n*-hexane, and the resulting organic extracts were evaporated to dryness. An internal standard, cholestanol (1 µg) (Sigma-Aldrich, Tokyo, Japan) was added to the dried extracts. The mixture was then incubated with *N*,*O*-bis(trimethylsilyl)trifluoroacetamide (15 µL) (Wako, Osaka, Japan) and *n*-hexane (100 µL) at 65 ${}^{\circ }$C overnight. The resulting solution was evaporated to dryness and dissolved in *n*-hexane (100 µL). The solutions were analysed by gas chromatography–mass spectrometry (GC-MS). The selective ion-monitoring mode was operated, and the area of the fragment ion peak or the molecular ion peak on the gas chromatogram was measured. The ion peaks for quantification are listed on Table 1. Since cholesterol is the product of the conversion, the intensity of enzyme activity was determined by the peak area ratio of detected cholesterol-d6 to desmosterol-d6. Although data varied in repeated experiments with different populations, the data showed similar tendencies. In the other experiments, the peak area ratios of targeted sterol to the internal standard were calculated instead.

### 2.6. GC-MS Conditions

GCMS-QP2010 Plus (Shimadzu, Kyoto, Japan) was operated with an electron ionization source at an electron energy of 70 eV in the positive mode for ion detection. A TR-5MS column (30 m × 0.25 mm i.d., 0.25 µm film thickness; Thermo Fisher Scientific, Franklin, MA, USA) was used for the analysis. The operating conditions were described as the following. Ion source temperature was set at 230 ${}^{\circ }$C. Interface temperature was set at 290 ${}^{\circ }$C. The column oven temperature was held at 80 ${}^{\circ }$C for 1 min after injection, then programmed to 240 ${}^{\circ }$C at 25 ${}^{\circ }$C/min, followed by 5 ${}^{\circ }$C/min to 290 ${}^{\circ }$C with a 10 min hold. Helium was used as the carrier gas at flow rate of 1.4 mL/min. The mass spectrum of the peak was recorded in the total ion current mode of the mass spectrometer with a mass range from *m/z* 45 to 600. The retention times of standard sterols were determined by analysing the TMS derivatives of commercial samples, including cholesterol-d7 (Cayman Chemical, Ann Arbor, MI, USA), desmosterol (Sigma Chemical Company, Japan) and desmosterol-d6 (Avanti, Alabaster, AL, USA) in GC-MS (Table 1). The retention times of TMS-cholesterol-d6 and TMS-7-dehydrodesmosterol were determined according to the corresponding peaks detected in the samples. The relative retention time was calculated to normalise the retention times of sterols in each sample. The identities of the detected sterols in each sample were confirmed with their relative retention times and mass spectra.

### 2.7. Examination of the Effects of Azacosterol on Enzyme Activity

Azacosterol solution was prepared by dissolving azacosterol in water. The incubation mixture was prepared as described in previous sections, but only the 15,300× *g*-pellet fraction was used. Incubation mixture containing different amounts of azacosterol (1, 5, 7.5, 10 and 25 ppm *w*/*v*) were then incubated, and sterols were extracted as described in above sections.

### 2.8. Preparation of cDNA

Two days after the female crickets emerged into adults, total RNA was extracted from crop, fat body, hindgut, anterior midgut, posterior midgut and Malpighian tubules using TRI-reagent (Molecular Research Centre, Inc., Cincinnati, OH, USA) according to the manufacturer’s protocol. The extracted RNA was incubated with RQ1 RNase-free DNase (Promega, Fitchburg, WI, USA) at 37 ${}^{\circ }$C for 1 h. RNA was then extracted by phenol and chloroform, followed by ethanol precipitation. cDNA was then synthesised using an oligo dT18 primer and reverse transcriptase, RevaTra Ace® (Toyobo, Osaka, Japan). The mixture was incubated at 42 ${}^{\circ }$C for 1 h, followed by incubation at 99 ${}^{\circ }$C for 5 min. In the experiment measuring the enzyme activity of ultracentrifugal fractions extracted from tissues, RNA was extracted with a slightly different method. After the preparation of incubation mixture for the examination of enzyme activity as described above, all the ultracentrifugal fractions were recombined. The partially combined fractions (50 µL) were added to TRI reagent (300 µL), and the extraction procedures were same as described above.

### 2.9. Examination of Transcriptional Levels of DHCR24 in Tissues

Quantitative polymerase chain reaction (qPCR) was performed with the cDNA derived in the previous section, THUNDERBIRD® SYBR Green (Toyobo, Osaka, Japan) and the primer sets in Table 2 on Thermal Cycler Dice Real Time System TP850 (TaKaRa, Shiga, Japan). The thermal cycling protocol of qPCR was as follows: initial denaturation at 95 ${}^{\circ }$C for 1 min and followed by 45 cycles of amplification. Each cycle consisted of 95 ${}^{\circ }$C for 10 s and 60 ${}^{\circ }$C for 30 s. The qPCR programme ended with dissociation curve analysis in the following order: 95 ${}^{\circ }$C for 15 s, 60 ${}^{\circ }$C for 30 s and 95 ${}^{\circ }$C for 15 s. The absolute quantity of each gene was derived from the second Derivative Maximum Method. Both the transcription levels of GbDHCR24-1 and GbDHCR24-2 were normalized to the that of β-actin. All datapoints were performed twice to confirm the reproducibility.

### 2.10. RNA Interference (RNAi)

The T-vector subcloned cDNA was amplified by using primers containing an additional T7 promotor sequence at the 5’-terminal (Table 2) under the following amplification conditions: initial denaturation at 96 ${}^{\circ }$C for 2 min, 40 cycles of amplification (94 ${}^{\circ }$C for 30 s, 55 ${}^{\circ }$C for 30 s and 72 ${}^{\circ }$C for 1 min) and followed 72 ${}^{\circ }$C for 5 min. RNA was then synthesised with T7 RNA polymerase using 800 ng of PCR products as template DNA. The synthesized RNA was purified by phenol/choloroform extraction and ethanol precipitation. RNA was dissolved in diethyl pyrocarbonate (DEPC)-treated water. RNA was diluted to a concentration of 3 µg/µL. The complementally synthesized RNA was denatured at 100 ${}^{\circ }$C for 5 min and was gradually cooled down to room temperature to produce dsRNA. The prepared dsRNA solution (6 µL) was injected into crickets. DsRed, which was prepared as previously described [19], was also injected into the crickets as a control. Three days after injection, the efficiency of RNAi and enzyme activity was examined by qPCR.

### 2.11. Statistical Analyses

The Mann–Whitney U test was used as the statistic tests where it was appropriate in this paper. In all the tests, *p* < 0.05 was considered as statistically significant. All experiments were repeated multiple times with different populations of crickets, and similar trends were confirmed in all the repeated trials.

## 3. Results

### 3.1. Characterization of GbDHCR24

**Figure 1:**
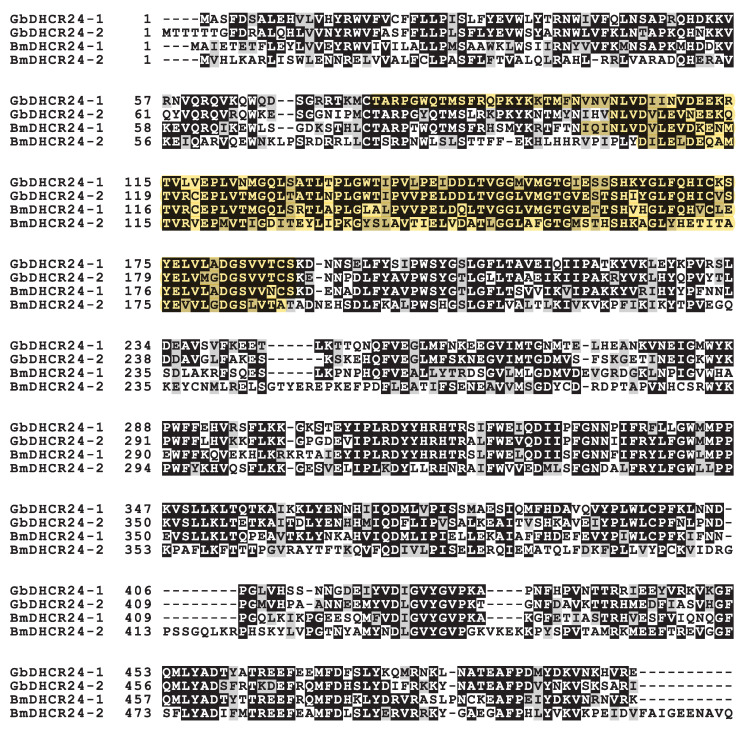
Alignment of *G. bimaculatus* DHCR24s (GbDHCR24-1 and GbDHCR24-2) and *B. mori* DHCR24 (BmDHCR24-1 and BmDHCR24-2). Identical amino acids at the same position are shaded in black, and similar amino acids at the same position are shaded in pale grey. The region matching the FAD binding domain on the Pfam database is highlighted in yellow.

**Figure 2:**
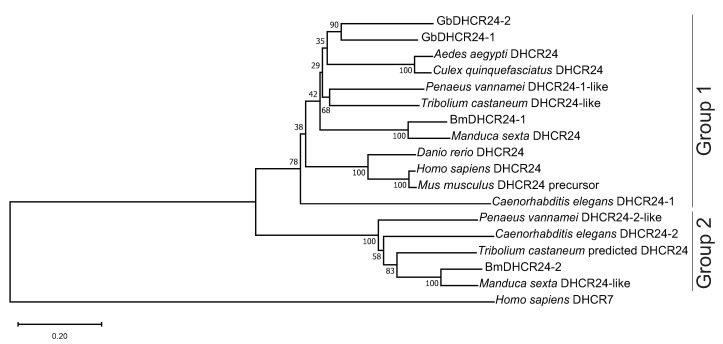
A phylogenetic tree showing the relationship between GbDHCR24 and DHCR24 of other species. Sequences of DHCR24s and related proteins were analysed phylogenetically using the Neighbour Joining method with MEGA X. The numbers next to the branches indicate that the percentage of the same branch was observed in the repeating construction of the phylogenetic tree. The details of the sequences used in the phylogenetic analysis are stated in Table S1. 7-dehydrocholesterol reductase is used as an outgroup in the analysis. The DHCR24 amino acid sequences were split into group 1 and 2.

In order to identify the homologous cDNAs encoding DHCR24 in *G. bimaculatus*, we searched the cDNA sequences based on the *B. mori* DHCR24 two homologues (BmDHCR24-1 and BmDHCR24-2) in our in-house database from RNA-sequencing analyses [13,14]. The BLAST search resulted in two nucleotide sequences encoding DHCR24 homologues in *G. bimaculatus* DHCR24, GbDHCR24-1 and GbDHCR24-2. cDNA sequences encoding GbDHCR24-1 and GbDHCR24-2 contained open reading frames of 1506 bp and 1515 bp, respectively. The deduced amino acid sequences were composed of 501 and 504 amino acids, respectively (Figure 1). The amino acid sequence of GbDHCR24-1 showed 56% and 39% identities to the amino acid sequences of BmDHCR24-1 and BmDHCR24-2, respectively. The amino acid sequence of GbDHCR24-2 showed 58% and 39% identities to the amino acid sequences of BmDHCR24-1 and BmDHCR24-2, respectively. The amino acid sequence of GbDHCR24-1 matched 66% of the sequence of GbDHCR24-2. Likewise, in the amino acid sequences of BmDHCR24s, the Pfam database showed that both GbDHCR24-1 and GbDHCR24-2 sequences of crickets contained flavin adenine dinucleotide (FAD) binding domain [20]. Next, we analysed the phylogenetic relationship between GbDHCR24s and the DHCR24 of the other species, including human, mouse and other insects (Figure 2). Using the distantly related 7-dehydrocholesterol reductase as an outgroup, the phylogenetic tree showed two distinct groups. Both GbDHCR24s clustered with group one, which also contained BmDHCR24-1 and the vertebrate DHCR24s. Group two was mostly consisted of DHCR24 sequences of non-vertebrate species.

### 3.2. Tissue Distribution of GbDHCR24 Expression

**Figure 3:**
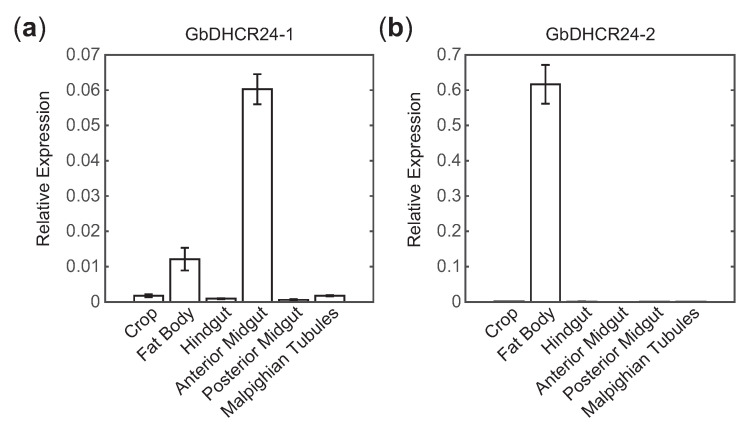
Tissue distribution of GbDHCR24s. Relative transcriptional level of (**a**) GbDHCR24-1 and (**b**) GbDHCR24-2 to β-actin in the two-spotted crickets (*n* = 6–8). Data are presented as mean ± SEM.

In order to analyse the expression sites of GbDHCR24s in crickets, quantitative real-time polymerase chain reaction (qPCR) was performed to measure the transcriptional levels of GbDHCR24s in tissues (crop, fat body, hindgut, anterior midgut, posterior midgut and Malpighian tubules). The highest transcriptional level of GbDHCR24-1 was observed in the anterior midgut (Figure 3a). On the other hand, the transcriptional level of GbDHCR24-2 was the highest in fat body (Figure 3b).

### 3.3. Examination of Enzyme Activity of GbDHCR24

**Figure 4:**
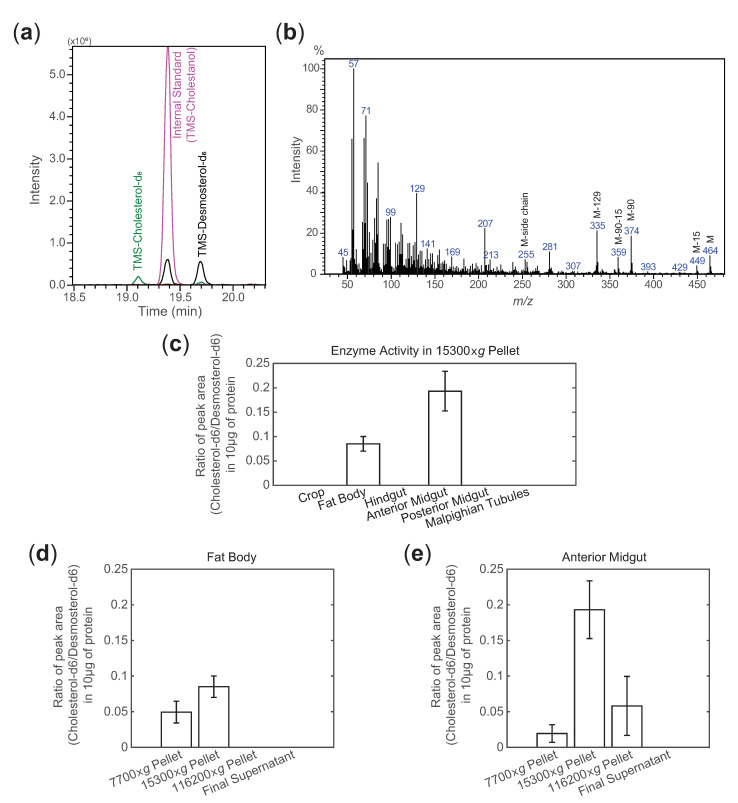
Examination of DHCR24 activity in fat body and anterior midgut. (**a**) Chromatogram of parent ions of TMS-cholesterol-d6 (*m/z* 464), TMS-cholestanol as an internal standard (*m/z* 460) and TMS-desmosterol-d6 (*m/z* 462). (**b**) Mass spectrum of cholesterol-d6 detected in the reacted mixture. The parent ion of cholesterol-d6 is at *m/z* 464 (M) and the characteristic fragment ions, at *m/z* 449 (M-15), 374 (M-90), 359 (M-90-15) and 335 (M-129), are derived by the loss of trimethylsilanol (TMSOH) (M-90), loss of methyl group (M-15), loss of both TMSOH and methyl group (M-90-15) and loss of the characteristic of 3β-hydroxy-5-ene steroids (M-129), respectively (Table S2). (**c**) The enzyme activity in the fraction that exhibited the strongest enzyme activity in different tissues. (**d**,**e**) The intensity of GbDHCR24 activity in fat body and anterior midgut was measured by the peak area ratio of cholesterol-d6 to desmosterol-d6 in four ultracentrifugal fractions, 7700× *g*-pellet fraction, 15,300× *g*-pellet fraction, 116,200× *g*-pellet fraction and final supernatant (*n* = 5–12). Data are presented as mean ± SEM.

In order to measure the enzyme activity in crickets effectively, we prepared homogenates of tissues, and they were separated into four fractions (7700× *g*-pellet fraction, 15,300× *g*-pellet fraction, 116,200× *g*-pellet fraction and the final supernatant) before the investigation. In order to trace the desmosterol-to-cholesterol-conversion, desmosterol-d6 was incubated with different ultracentrifugal fractions. A peak corresponding to TMS-cholesterol-d6 was detected when the desmosterol-to-cholesterol-conversion was successful (Figure 4a). The mass spectrum and the relative retention times of TMS-cholesterol-d6 were confirmed by comparing with the data obtained from the TMS derivatives of commercially available cholesterol and cholesterol-d7 (Figure 4b and Figure S1; Table S2). The conversion was observed in at least one fraction extracted from fat body or anterior midgut. No TMS-cholesterol-d6 was detected in any fractions extracted from crop, posterior midgut, hindgut and Malpighian tubules (Figure 4c). Among the fractions, the reactions using the 15,300× *g*-pellet fraction of both fat body and anterior midgut gave the highest ratio of cholesterol-d6 to desmosterol-d6 (Figure 4d,e). Smaller amounts of cholesterol-d6 were also observed in the 7700× *g*-pellet fraction and the 116,200× *g*-pellet fraction of both tissues. Almost none of the converted product was found in the final supernatant. This indicated that the 15,300× *g*-pellet fraction was the predominant fraction for the detection of DHCR24 activity. Hence, we used 15,300× *g*-pellet fraction extracted from the tissue homogenates for the evaluation of GbDHCR24 activity in crickets in later experiments.

### 3.4. Effects of Azacosterol on Crickets

**Figure 5:**
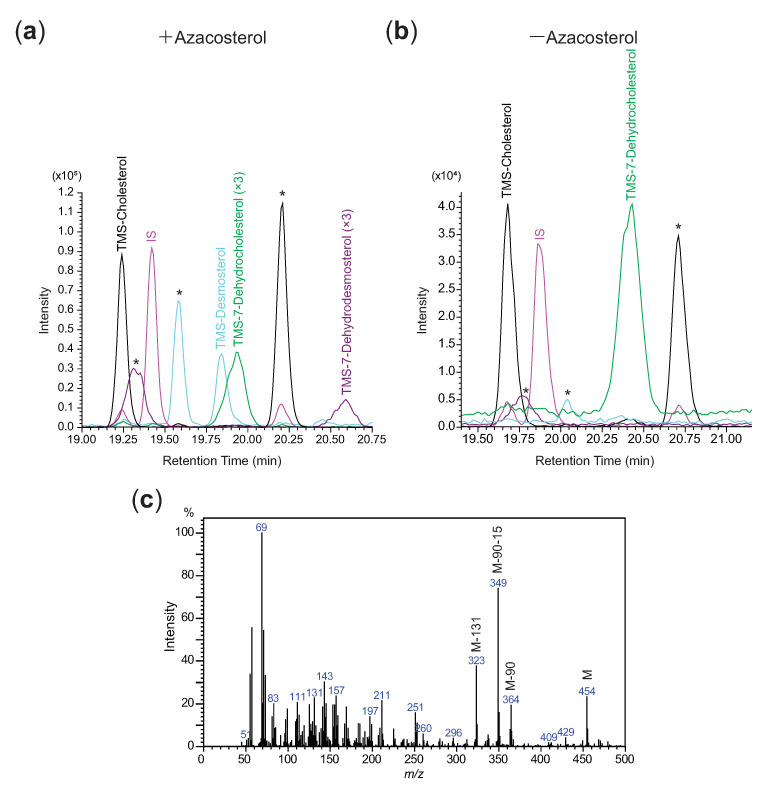
Chromatogram of ions corresponding to TMS-desmosterol (*m/z* 343) and TMS-7-dehydrodesmosterol (*m/z* 349) detected in anterior midgut of (**a**) crickets fed on diet containing azacosterol and that of (**b**) crickets fed on diet without azacosterol. The number of times the peak was magnified is indicated in the bracket next to the sterol name. (**c**) Mass spectrum of TMS-7-dehydrodesmosterol detected in all anterior midgut samples of crickets in this study (*n* = 7). The * indicates the peaks of sterols (Unidentified sterol (RRT = 1.005), TMS-5α-cholesta-7,22-dien-3β-ol (RRT = 0.992) and TMS-lathosterol (RRT = 0.961) that were not studied in this paper.

In order to examine if only DHCR24 would contribute to the desmosterol-to-cholesterol-conversion, azacosterol (a specific DHCR24 inhibitor) was introduced to crickets. The effect of azacosterol was first examined in vitro. When different amounts of azacosterol were incubated with the 15,300× *g*-pellet fraction, the conversion of desmosterol-to-cholesterol was inhibited in a dose-dependent manner (Figure S2). In the anterior midgut, 5 ppm of azacosterol (*w*/*v*) was adequate to fully inhibit DHCR24 (Figure S2a). The effect of azacosterol was also examined in vivo via feeding crickets the diet containing azacosterol. As a result, higher abundance of desmosterol was detected in fat body and anterior midgut in the crickets fed on diets containing 200 ppm (*w*/*w*), while almost no desmosterol was detected in crickets fed on diet without azacosterol (Figure 5a and Figure S3). In addition, a peak corresponding to 7-dehydrodesmosterol, which was identified after comparing with the published mass spectrum, was also detected in all samples of anterior midgut in vivo (*n* = 8) (Figure 5b,c) [21].

### 3.5. Effects of Knockdown of GbDHCR24s

**Figure 6:**
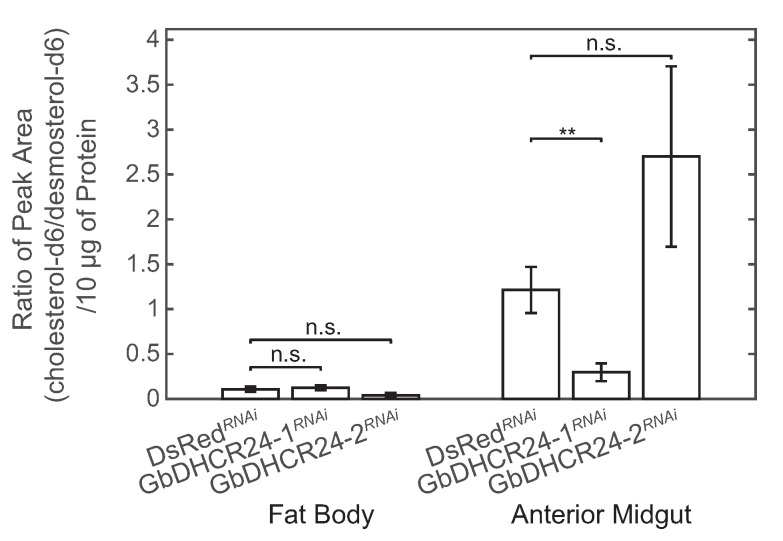
Enzyme activity of the 15,300× *g*-pellet fraction extracted from fat body and anterior midgut of GbDHCR24 knockdown crickets (*n* = 6). The changes in enzyme activity between the DsRed${}^{RNAi}$ and GbDHCR24${}^{RNAi}$ crickets were analysed by the Mann–Whitney U test. The significance is indicated by ** p < 0.01. n.s. indicates no significance between groups. Data are presented as mean ± SEM.

In order to examine the contribution of each variant to the desmosterol-to-cholesterol-conversion, we measured DHCR24 activity in fat body and anterior midgut after performing RNA interference (RNAi) through the injection of dsRNA. The efficiency of knockdown of GbDHCR24-1 and GbDHCR24-2 was measured by qPCR (Figure S4). GbDHCR24-1 knockdown reduced the transcriptional level of GbDHCR24-1 by 48% and 79% in fat body and anterior midgut, respectively. Meanwhile, GbDHCR24-2 knockdown reduced the transcriptional level of GbDHCR24-2 in fat body and anterior midgut by 83% and 46%, respectively. The knockdown of GbDHCR24-1 in anterior midgut and the knockdown of GbDHCR24-2 in fat body were considered as successful knockdowns by the statistic test. In the tissues which the transcriptional levels of GbDHCR24s were successfully affected by RNAi, GbDHCR24-1 activity in anterior midgut samples extracted from the GbDHCR24-1${}^{RNAi}$ crickets was reduced by 76%. The statistic tests considered there was a significant reduction in enzyme activity in anterior midgut derived from GbDHCR24-1${}^{RNAi}$ crickets. On the other hand, DHCR24 activity in fat body of GbDHCR24-2${}^{RNAi}$ crickets was reduced by 63%, but the statistic test did not suggest that knockdown of GbDHCR24s affect the enzyme activity in fat body (Figure 6).

## 4. Discussion

We successfully identified the GbDHCR24 sequences and demonstrated GbDHCR24 activities in crickets. GbDHCR24-1 seemed to be the homologue that resulted in the production of cholesterol from desmosterol, but the function of GbDHCR24-2 remained unknown. In the amino acid sequences of GbDHCR24, the characteristic FAD binding domain was found in each variant, which is consistent with the findings in human DHCR24 and BmDHCR24s. As cholesterol is produced from desmosterol by DHCR24 only in the presence of nicotinamide adenine dinucleotide 2’-phosphate (NADPH), the conserved FAD binding domain might be used for NADPH binding to DHCR24 [3,13,15,22].

Another finding about GbDHCR24 is the potential subcellular location of GbDHCR24, which is also consistent with DHCR24 of the other species. When the enzyme activities of different ultracentrifugal fractions were examined, the 15,300× *g*-pellet fraction showed the strongest activity (Figure 4c,d). This fraction might contain ER and Golgi apparatus as elsewhere reported that these two organelles could be obtained under similar centrifuge conditions [23,24]. Since both ER and Golgi apparatus are associated with DHCR24, strong DHCR24 activity was detected in the ultracentrifugal fraction containing these two organelles [25,26]. The desmosterol-to-cholesterol–conversion was also detected in other ultracentrifugal fractions, but they were more likely resulted from incomplete isolation of organelles (Figure 4).

Despite GbDHCR24s sharing some similarities with the other DHCR24s, GbDHCR24s demonstrated their unique features. The phylogenetic tree showed that DHCR24 either exists as (1) a single variant that belongs to group one, such as the human DHCR24; or (2) two variants that do not cluster in the same group, but the enzyme activities of the two variants are different, which was proved to be the case in *B. mori* [14]. However, the situation in the currently identified GbDHCR24s is neither of the cases described above. The sequences of the two GbDHCR24 variants match 66% identity, and the two variants cluster in the same clade. Moreover, these variants have different enzyme activities. The data showed that the conversion of desmosterol to cholesterol is more likely faciliated by GbDHCR24-1 but not GbDHCR24-2 (Figure 3).

In the RNAi experiment that investigated the enzyme activity of each variant, the data showed that no matter which variant was knocked down, the conversion of desmosterol in fat body was not affected. Presuming that both GbDHCR24-1 and GbDHCR24-2 can facilitate the desmosterol-to-cholesterol-conversion, when one variant was knocked down, the conversion could still be facilitated by the unaffected variant. Moreover, the enzyme activity in fat body appeared to be much weaker than that in anterior midgut. The effect of knockdown of one variant in fat body might not show as drastically as it could be in anterior midgut. This did not happen in the anterior midgut because the transcriptional level of GbDHCR24-2 in anterior midgut was almost zero. Even if GbDHCR24-2 functions, the influence would be too small to be detected.

Although GbDHCR24-1 and GbDHCR24-2 clustered in the same clade on the phylogenetic tree, the transcriptional level and enzyme activity in each tissue varied. The differences may be derived from variations in the amino acid sequences. In humans, it was suggested that the mutation of the phosphorylation sites that lied within and outside the FAD binding domain could result in the loss of enzyme activity [27]. Based on the predictions of phosphorylation sites, GbDHCR24-1 and GbDHCR24-2 seemed to have different phosphorylation sites (Figure S5) [28]. Moreover, GbDHCR24-1 appeared to have more tyrosine phosphorylation sites. It is uncertain if the number of phosphorylation sites and the type of phosphorylation sites affect the functions of GbDHCR24.

Another interesting observation is the tissue distribution of the GbDHCR24. Although all examined tissues expressed at least one variant of GbDHCR24s, DHCR24 activity was only detected in fat body and anterior midgut, and the transcriptional levels of GbDHCR24-2 in these two tissues were much higher than those detected in the other examined tissues (Figure 3 and Figure 4c,d). The enzyme activity in other tissues may be too weak to be detected. It is unclear if there is any relationship between the transcription level and the intensity of the enzyme activity. However, we also have to consider other factors, for example, the efficiency of translation and the regulatory effects of metabolites as we mentioned above [29,30].

In order to confirm if GbDHCR24 is the only enzyme contributing to the desmosterol-to-cholesterol-conversion, an inhibitor of DHCR24, azacosteterol, was added to the diets of crickets [31,32]. The addition of azacosterol in diets can result in accumulation of desmosterol in vitro and in vivo, as stated in the previously published researches conducted on mammals and insects [32,33,34]. As what we had predicted, diet coated with azacosterol caused the accumulation of desmosterol in fat body and anterior midgut in crickets by inhibiting DHCR24 activity (Figures S2 and S3). This indicated that GbDHCR24 is the only enzyme that can convert desmosterol to cholesterol in crickets. The observation is consistent with the findings in *Manduca sexta* larvae after feeding on a diet containing azacosterol ranging from 16.5 ppm to 260 ppm wet weight and β-sitosterol at 260 ppm wet weight over 20 days [7,35]. All tested concentrations of azacosterol seemed to have caused impairment of DHCR24 activity.

Apart from the desmosterol-to-cholesterol-conversion, GbDHCR24 might also facilitate reduction of another sterol substrate. It has been reported that reactions of DHCR24 also include the conversion from 7-dehydrodesmosterol to 7-dehydrocholesterol and the conversion from 5α-cholesta-7,24-dien-3β-ol to lathosterol [36]. In all anterior midgut samples collected from crickets fed with diets containing azacosterol, a peak corresponding to 7-dehydrodesmosterol was detected (Figure 5). Azacosterol might have inhibited the conversion from 7-dehydrodesmosterol to 7-dehydrocholesterol. Indeed, 7-dehydrocholesterol was detected in all anterior midgut samples, even in the crickets that fed on diet without azacosterol. 7-dehydrocholesterol was probably a product of the conversion facilitated by GbDHCR24.

In conclusion, we demonstrated that the two-spotted cricket possessed two homologues of DHCR24, GbDHCR24-1 and GbDHCR24-2. We confirmed that GbDHCR24-1 in the two-spotted cricket can convert desmosterol to cholesterol mainly in anterior midgut. The present data also showed that GbDHCR24 seemed to be the only enzyme that can convert desmosterol to cholesterol in the two-spotted cricket. The present data indicated that omnivorous insects might be able to utilise phytosterols to meet their cholesterol requirement via the desmosterol-to-cholesterol-conversion as a stepping stone. Additionally, the discovery of the new intermediate may reveal more clues on the sterol conversion pathway in the two-spotted cricket.

## Figures and Tables

**Table 1:** List of sterols analysed.

Sterols	Parent Ion	Peak for Identification	Relative Retention
(TMS Derivatives)	(*m/z*)	(*m/z*)	Time (RRT)
Cholesterol	458	458	1.009
Cholesterol-d6	464	464	1.013
Cholesterol-d7	465	465	1.014
Desmosterol	456	343	0.978
Desmosterol-d6	462	462	0.984
7-Dehydrocholesterol	456	325	0.974
7-Dehydrodesmosterol	454	349	0.943

**Table 2:** Primers used in this research.

Experiment	Primer	Sequence (5′ to 3′)
qPCR	DHCR24-1 Forward	AACAATGATCCAGGCCTCGT
	DHCR24-1 Reverse	AGGAACGCCGTATACACCAA
	DHCR24-2 Forward	ATGGGTGTTCGCGTCATTCT
	DHCR24-2 Reverse	TGAAGACCAACCAGTTGCGT
	β-actin Forward	TTGACAATGGATCCGGAATGT
	β-actin Reverse	AAAACTGCCCTGGGTGCAT
RNAi	T7-DHCR24-1 Forward	GCTTCTAATACGACTCACTATAGCGATAAGAAGGTGCGCAACG
	T7-DHCR24-1 Reverse	GCTTCTAATACGACTCACTATAGTAACGACACTGCCATCTGCC
	T7-DHCR24-2 Forward	GCTTCTAATACGACTCACTATAGATGCCTCCCAAGGTTTCGTT
	T7-DHCR24-2 Reverse	GCTTCTAATACGACTCACTATAGACACGTCAGGAAAAGCCTCC

## Data Availability

Not applicable.

## Supplementary Materials

The following tables and figures are available online https://www.mdpi.com/article/10.3390/insects12090782/s1, Table S1: Details of sequences used in the phylogenetic analysis, Table S2: Fragmentation of TMS derivatives of cholesterol, cholesterol-d7 and cholesterol-d6, Figure S1: Mass spectra of TMS derivatives of commercial samples of cholesterol and cholesterol-d7, Figure S2: Effects of different amounts of azacosterol on the enzyme activity in the 15,300× *g*-pellet fraction extracted from fat body and anterior midgut, Figure S3: Effects of azacosterol on the amount of desmosterol in fat body and anterior midgut, Figure S4: The efficiencies of the knockdown of GbDHCR24s, Figure S5: The alignment of GbDHCR24 showing the predicted phosphorylation sites.
