# Supplementary material for: Identification and Characterization of 24-Dehydrocholesterol Reductase (DHCR24) in the Two-Spotted Cricket, Gryllus bimaculatus
Source: Insects. 2021 Sep 1;12(9):782. doi: 10.3390/insects12090782 (PMC8471071; doi:10.3390/insects12090782)
Supplement: Supplementary file 1 [file insects-12-00782-s001.zip › insects-1339289-supplementary.pdf]

Table S1: Details of sequences use in the phylogenetic analysis.

Species	Description on Figure 2	Registered Name of Enzyme on NCBI	NCBI Accession Number	Number of Amino Acids
Aedes aegypti	DHCR24	delta(24)-sterol reductase	XP_001655874.2	504
Bombyx mori	BmDHCR24-1	delta(24)-sterol reductase	XP_004926865.1	506
Bombyx mori	BmDHCR24-2	delta(24)-sterol reductase	XP_004926210.1	531
Caenorhabditis elegans	DHCR24-1	Delta(24)-sterol reductase	NP_001362099.1	516
Caenorhabditis elegans	DHCR24-2	Delta(24)-sterol reductase	NP 510594.1	538
Culex quinquefasciatus	DHCR24	24-dehydrocholesterol reductase	EDS41303.1	504
Danio rerio	DHCR24	Delta(24)-sterol reductase	NP_001008645.1	516
Homo sapiens	DHCR24	24-dehydrocholesterol reductase	AAH11669.1	516
Manduca sexta	DHCR24	delta(24)-sterol reductase	XP_030025267.2	507
Manduca sexta	DHCR24-like	delta(24)-sterol reductase-like	XP_037295187.1	533
Mus musculus	DHCR24 precursor	delta(24)-sterol reductase precursor	NP_444502.2	516
Tribolium castaneum	DHCR24-like	PREDICTED: delta(24)-sterol reductase-like isoform X1	XP_001810773.1	505
Tribolium castaneum	predicted DHCR24	PREDICTED: delta(24)-sterol reductase	XP_966520.1	525
Penaeus vannamei	DHCR24-1 like	delta(24)-sterol reductase-like	XP 027224655.1	504
Penaeus vannamei	DHCR24-2 like	delta(24)-sterol reductase-like	XP 027238556.1	537
Homo sapiens	DHCR7	7-dehydrocholesterol reductase	NP_001157289.1	475

Table S2: Fragmentations of TMS derivatives of cholesterol, cholesterol-d7 and cholesterol-d6.

Annotation	Ions in TMS-Cholesterol (*m*/*z*)	Ions in TMS-Cholesterol-d7 (*m*/*z*)	Ions in TMS-Cholesterol-d6 (*m*/*z*)
M	458	465	464
M-CH_3	443	450	449
M-TMSOH	368	375	374
M-TMSOH-CH_3	353	360	359
M-C_1,C_2,C_3-TMSO	329	336	335
M-C_1,C_2,C_3-TMSO-CH_2CH_2	301	308	307
C_1,C_2,C_3+TMSO	129	129	129

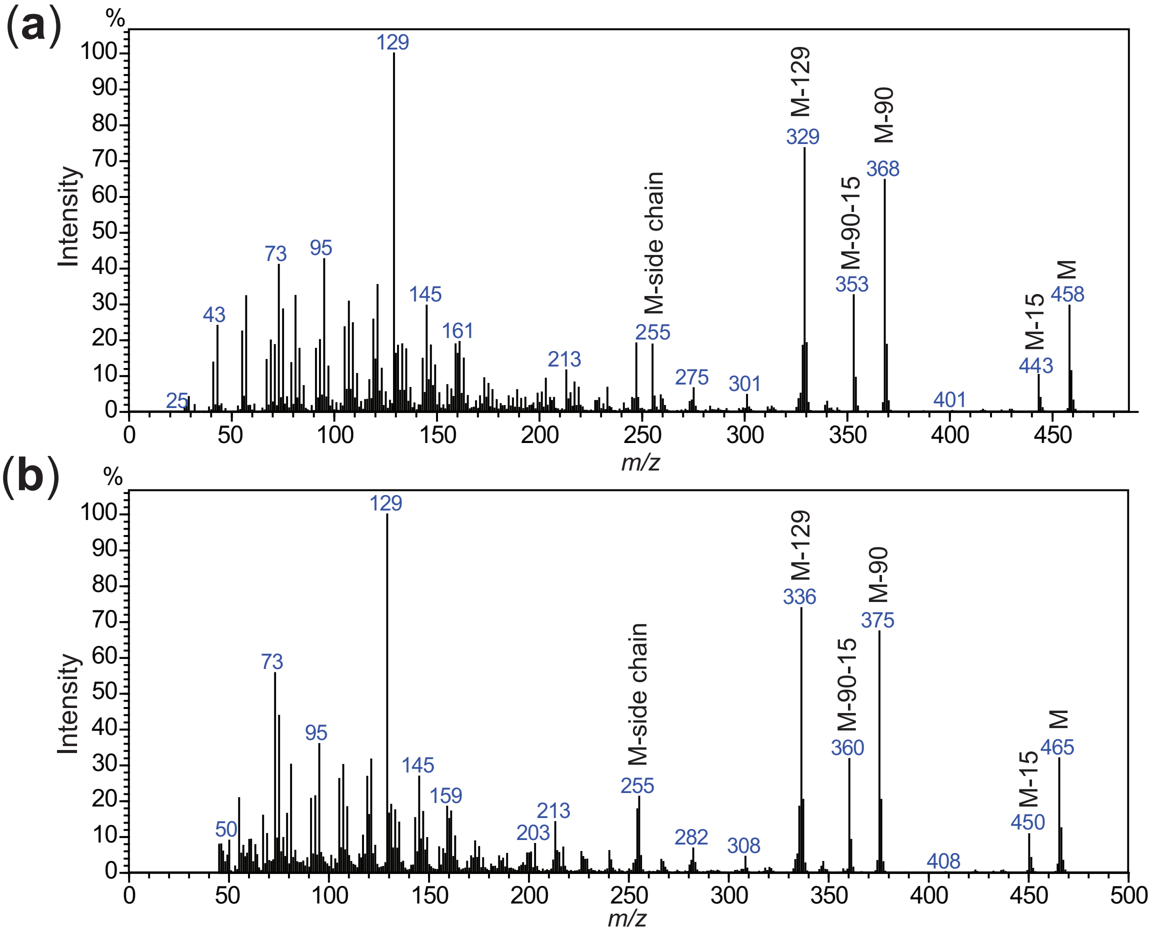

Figure S1: Mass spectra of TMS derivatives of commercial samples of (**a**) cholesterol and (**b**) cholesterol-d7.

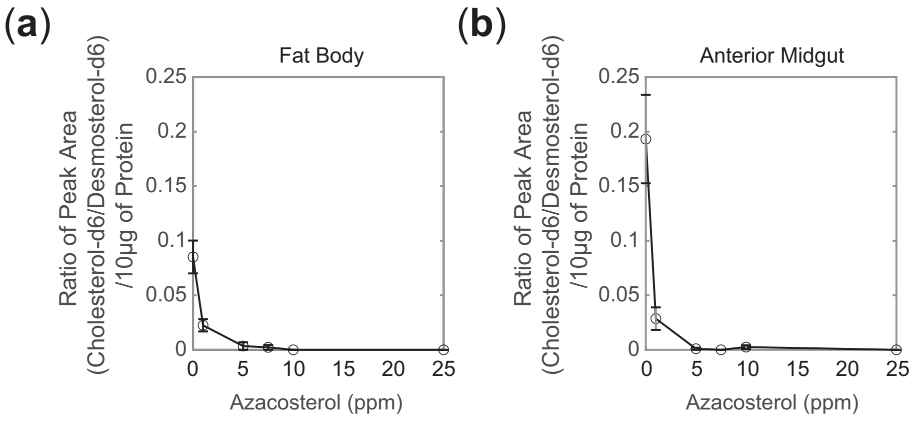

Figure S2: Effect of different amounts of azacosterol on the enzyme activity in the 15300×g-pellet fraction extracted from (**a**) fat body and (**b**) anterior midgut n = 6-15). Data is presented as mean±SEM.

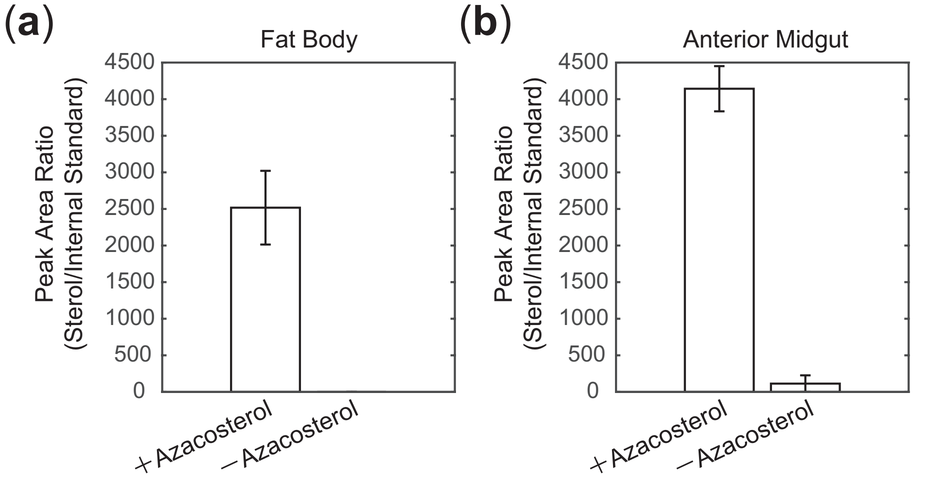

Figure S3: Effect of azacosterol on the amount of desmosterol in (**a**) fat body and (**b**) anterior midgut. Amount of desmosterol were standardized by the total amount of RNA. Data was derived from crickets feeding on diet with and without azacosterol for 18 hours (n = 7). Data is presented as mean±SEM.

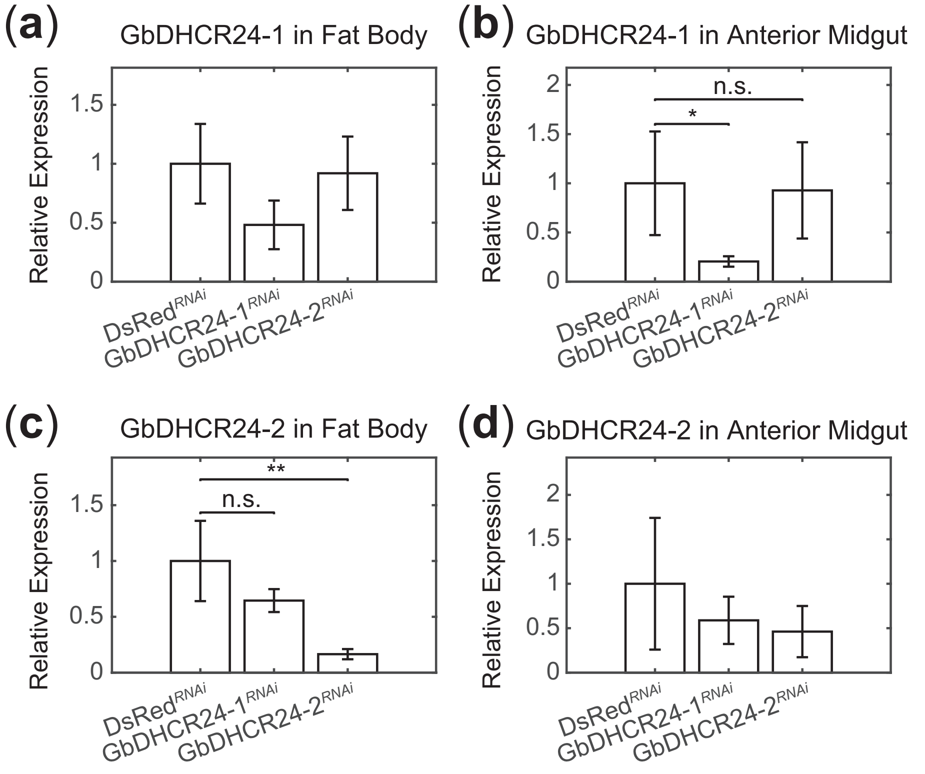

Figure S4: The efficiencies of the knockdown of GbDHCR24s were evaluated by qPCR. The average of relative expression levels of each GbDHCR24 to β-actin in the DsRed^{RNAi} crickets was standardized to 1. In order to normalise the data, the relative expression levels of GbDHCR24-1 to β-actin in the GbDHCR24^{RNAi} crickets were divided by the average of relative expression levels of each GbDHCR24 variant to β-actin in the DsRed^{RNAi} crickets. (n = 6). (**a, b**) Relative expression of GbDHCR24-1. (**c,d**) Relative expression of GbDHCR24-2. Data is presented as mean±SEM. Mann-Whitney U test was performed to analyse the statistical differences between the results obtained from DsRed^{RNAi} and GbDHCR24^{RNAi}. * $P < 0.05$ and ** $P < 0.01$.

```
GbDHCR24-1     1  ----MASFDSALEHVLVHYRWVFVCFFLLPISLFYEVWLYTRNWIVFQLNSAPRQHDKKV
GbDHCR24-2     1  MTTTTTGFDRALQHLVVNYRWVFASFFLLPLSFLYEVWSYARNWLVFKLNTAPKQHNKKV

GbDHCR24-1    57  RNVQRQVKQWQDSGRRTKMCTARPGWQTMSFRQPKYKKTMFNVNVNLVDIINVDEEKRTV
GbDHCR24-2    61  QYVQRQVRQWKESGGNIPMCTARPGYQTMSLRKPKYKNTMYNIHVNLVDVLEVNEEKQTV

GbDHCR24-1   117  LVEPLVNMGQLSATLTPLGWTIPVLPEIDDLTVGGMVMGTGIESSSHKYGLFQHICKSYE
GbDHCR24-2   121  RCEPLVTMGQLTATLNPLGWTIPVVPELDDLTVGGLVMGTGVESTSHIYGLFQHICVSYE

GbDHCR24-1   177  LVLADGSVVTCSKDNNSELFYSIPWSYGSLGFLTAVEIQIIPATKYVKLEYKPVRSLDEA
GbDHCR24-2   181  LVMGDGSVVTCSKENNPDLFYAVPWSYGTLGLLTAAEIKIIPAKRYVKLHYQPVYTLDDA

GbDHCR24-1   237  VSVFKEETLKTTQNQFVEGLMFNKEEGVIMTGNMTELHEANKVNEIGMWYKPWFFEHVRS
GbDHCR24-2   241  VGLFAKES-KSKEHQFVEGLMFSKNEGVIMTGDMVSFSKGETINEIGKWYKPWFFLHVKK

GbDHCR24-1   297  FLKKGKSTEYIPLRDYYHRHTRSIFWEIQDIIPFGNNPIFRFLLGWMMPPKVSLLKLTQT
GbDHCR24-2   300  FLKKGPGDEVIPLRDYYHRHTRALFWEVQDIIPFGNNIIFRYLFGWMMPPKVSLLKLTET

GbDHCR24-1   357  KAIKKLYENNHIIQDMLVPISSMAESIQMFHDAVQVYPLWLCPFKLNNDPGLVHSSNNGD
GbDHCR24-2   360  KAITDLYENHHMIQDFLIPVSALKEAITVSHKAVEIYPLWLCPFNLPNDPGMVHPAANNE

GbDHCR24-1   417  EIYVDIGVYGVPKAPNFHPVNTTRRIEEYVRKVKGFQMLYADTYATREEFEEMFDFSLYK
GbDHCR24-2   420  EMYVDLGVYGVPKTGNFDAVKTTRHMEDFIASVHGFQMLYADSFRTKDEFRQMFDHSLYD

GbDHCR24-1   477  QMRNKLNATEAFPDMYDKVNKHVRE
GbDHCR24-2   480  IFRKKYNATEAFPDVYNKVSKSARI
```

Figure S5: The alignment of GbDHCR24 showing the predicted phosphorylation sites. The phosphorylation sites predicted by NetPhos are shaded in blue. Identical amino acids at the same position are shaded in black, and similar amino acids at the same position are shaded in pale grey. The region matching the FAD binding domain on the Pfam database is indicated by the red line.
